# The TPR-containing domain within Est1 homologs exhibits species-specific roles in telomerase interaction and telomere length homeostasis

**DOI:** 10.1186/1471-2199-12-45

**Published:** 2011-10-18

**Authors:** David CF Sealey, Aleksandar D Kostic, Catherine LeBel, Fiona Pryde, Lea Harrington

**Affiliations:** 1 Department of Medical Biophysics, University of Toronto; Campbell Family Institute for Breast Cancer Research and Ontario Cancer Institute, University Health Network, Toronto, Ontario, Canada; 2 Wellcome Trust Centre for Cell Biology, University of Edinburgh, Edinburgh, UK; 3 Department of Pathology, Harvard Medical School, Boston, MA, USA; 4 Samuel Lunenfeld Research Institute, Mount Sinai Hospital, Toronto, Ontario, Canada; 5 Université de Montréal, Institute de Recherche en Immunologie et en Cancérologie, Montréal, Québec, Canada

## Abstract

**Background:**

The first telomerase-associated protein (Est1) was isolated in yeast due to its essential role in telomere maintenance. The human counterparts EST1A, EST1B, and EST1C perform diverse functions in nonsense-mediated mRNA decay (NMD), telomere length homeostasis, and telomere transcription. Although Est1 and EST1A/B interact with the catalytic subunit of yeast and human telomerase (Est2 and TERT, respectively), the molecular determinants of these interactions have not been elaborated fully.

**Results:**

To investigate the functional conservation of the EST1 protein family, we performed protein-protein interaction mapping and structure-function analysis. The domain in hEST1A most conserved between species, containing a TPR (tricotetrapeptide repeat), was sufficient for interaction of hEST1A with multiple fragments of hTERT including the N-terminus. Two mutations within the hTERT N-terminus that perturb *in vivo *function (NAAIRS_92_, NAAIRS_122_) did not affect this protein interaction. ScEst1 hybrids containing the TPR of hEST1A, hEST1B, or hEST1C were expressed in yeast strains lacking *EST1*, yet they failed to complement senescence. Point mutations within and outside the cognate ScEst1 TPR, chosen to disrupt a putative protein interaction surface, resulted in telomere lengthening or shortening without affecting recruitment to telomeres.

**Conclusions:**

These results identify a domain encompassing the TPR of hEST1A as an hTERT interaction module. The TPR of *S. cerevisiae *Est1 is required for telomerase-mediated telomere length maintenance in a manner that appears separable from telomere recruitment. Discrete residues in or adjacent to the TPR of Est1 also regulate telomere length homeostasis.

## Background

In *S. cerevisiae*, telomeres are usually maintained by telomerase or *RAD52*-dependent recombination. The 'ever shorter telomere' genes *EST1*, *EST2 *(the telomerase reverse transcriptase, TERT), *EST3*, and *TLC1 *(the telomerase RNA) are essential for telomerase function because loss of any one gene results in progressive telomere shortening and senescence [1,2]. Mutation of *CDC13 *- an essential gene - elicits similar consequences [2,3]. Rare "survivors" can bypass this senescence by maintaining telomeres through recombination. The homologous recombination factor Rad52 is important for the generation of telomerase-independent Type I and Type II survivors in which telomeres are maintained by amplification of Y' elements or telomeric repeats, respectively; more rarely, survival can occur without Rad52 [4-10].

Cdc13 and Est1 are critical for the recruitment of the telomerase core complex (Est2-Tlc1) to telomeres in S phase [reviewed in [11-14]]. Cdc13 binds to single-stranded telomeric DNA [15,16] and associates with telomeres throughout the cell cycle, with a peak in association during S phase [17,18]. Est1 also binds to single-stranded telomeric DNA [19] and associates with telomeres in S phase [17,18,20]. Cdc13 and Est1 interact physically [21] and genetically, as evidenced by the unlinked complementation of *cdc13-2 *and *est1-60 *alleles, each of which affects telomere maintenance and viability [15,17,22,23]. Several findings suggest that Est1 recruits Est2 to the telomere in S phase by acting as an intermediary between Cdc13 and *TLC1*. For example, the telomere shortening and senescence that occur in the absence of *EST1 *are rescued by expression of a Cdc13-Est2 fusion protein [22]. Est1 binds to *TLC1 *[24-28], and the telomeric localization of Est2 in S phase is reduced when the region of *TLC1 *responsible for the Est1 interaction is deleted, or when *EST1 *is deleted [29].

In other organisms, Est1 homologs similarly associate with telomerase and regulate telomere length and stability, although in some instances their precise contributions to telomere function are still being uncovered. *S. pombe *Est1 associates with active telomerase in cell extracts, and *est1- *cells exhibit telomere shortening, senescence, and defects in chromosome end protection [30]. In *C. albicans*, telomere length in *est1Δ *cells fluctuates over serial passages, suggesting that Est1 may repress homologous recombination at telomeres [31]. In humans, three Est1 homologs, hEST1A/SMG6, hEST1B/SMG5 and hEST1C/SMG7 (hereafter referred to hEST1A, hEST1B and hEST1C) interact with chromatin and bind preferentially at telomeres [32-34]. Human EST1A and EST1B associate with active telomerase in cell lysates and *in vitro *[32,33]. Like ScEst1, hEST1A binds single-stranded telomeric DNA [33]. Transient over-expression (or depletion) of hEST1A causes telomere uncapping/end-to-end fusion and apoptosis, and stable over-expression of hEST1A in telomerase-positive cell lines elicits telomere shortening that can be mitigated by co-expression of hTERT [32-34].

Human EST1A, EST1B, and EST1C also possess functional homology to the *C. elegans *nonsense-mediated mRNA decay (NMD) factors SMG-6, SMG-5, and SMG-7 [32,33,35-38]. Transcripts containing premature termination codons (PTC) upstream of a terminal exon-exon junction are degraded by nonsense-mediated mRNA decay (NMD) - a process involving the phosphorylation and dephosphorylation of UPF1 by SMG1 and PP2A, respectively [reviewed in [39,40]]. The three EST1 proteins form complexes with SMG1, UPF1, PP2A, and other components of the NMD pathway [35,38], and mediate the dephosphorylation of UPF1 via recruitment of PP2A [35,38]. Depletion of hEST1A, hEST1B or hEST1C by RNA interference results in stabilization of PTC-containing mRNA [37,41,42]. Depletion of UPF1, SMG1, or hEST1A/SMG6 also leads to an increase in the intensity and number of foci containing the telomeric transcript TERRA [reviewed in [43]]. Parenthetically, the levels of *S. cerevisiae *Est1, Est2, Est3, Stn1, and Ten1 are regulated by NMD [44], but ScEst1 has no known role in NMD [42].

The complex functions of EST1 homologs are mirrored by their diversity in size and structure across species (Additional file 1, Figure S1). The region of highest homology among EST1 proteins includes tetratricopeptide repeat (TPR) consensus sequences [30-33] (Additional file 1, Figure S1). Typically, TPRs mediate protein-protein interactions [45]. The structure of hEST1C reveals a *bona fide *14-3-3-like TPR comprised of alpha helices; several contiguous upstream alpha helices fold into a TPR-like structure despite the fact they lack a TPR repeat consensus sequence [46]. Upstream of the TPR, hEST1A contains an N-terminal DNA binding activity [[33,42]; Jen Cruickshank and Lea Harrington, unpublished], and the N-terminus also interacts with hTR and other RNAs through an hTR-interaction domain (TRID) [47]. A region downstream of the hEST1B TPR resembles the DNA binding domain of ScEst1 [48], although DNA binding by hEST1B has not been described. Both hEST1A and hEST1B contain a C-terminal 'PilT N-terminal' (PIN) domain [32,33,36,49]. The PIN domain of hEST1A possesses a single-stranded RNA endonuclease activity and degrades PTC-containing mRNA [49,50]. The nuclease activity of the hEST1B PIN domain is greatly reduced likely due to the absence of critical residues in the active site [49]. This extensive cross-species complexity prompted us to conduct structure-function analysis of human and *S. cerevisiae *EST1 proteins using biochemical and genetic methods.

## Results

### Mapping the interactions between hEST1A and hTERT

**Figure 1:**
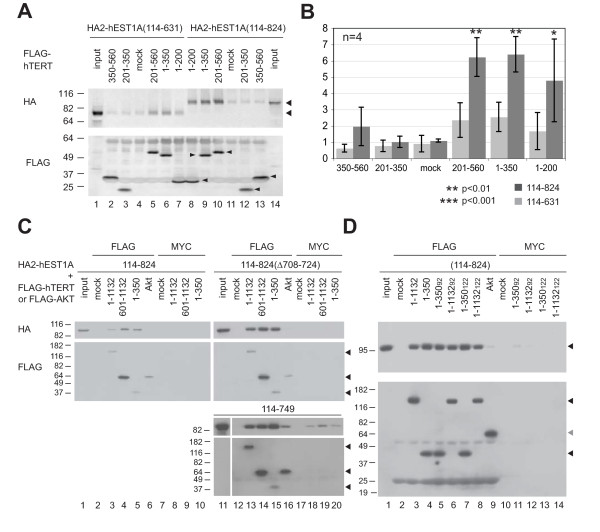
**The human EST1A TPR is sufficient for interaction with hTERT N- and C-termini**. (A) hEST1A(114-824) but not hEST1A (114-631) interacts with hTERT N-terminus independently of hTR. Proteins synthesized in RRL were precipitated onto anti-FLAG resin (mock reactions contained no cDNA), washed, and treated with micrococcal nuclease (MNase). The precipitate was subjected to western blotting using anti-HA (top) followed by anti-FLAG (bottom) antibodies. Input, 20% of input HA2-hEST1A. Molecular mass (kDa) is indicated. One of 4 independent experiments is shown. MNase activity was verified via electrophoresis of DNA (data not shown). (B) Quantification of data in Panel A. The levels of hEST1A(114-824) or hEST1A(114-631) associated with hTERT fragments (or mock; no plasmid) are expressed as the normalized hEST1A signal relative to the normalized hTERT signal (see Methods; average ± S.D., n = 4). P-values represent statistically significant enrichment above the mock control. (C) Upper: Putative disordered loop in hEST1A TPR is dispensable for the hTERT interaction. Experiment as in Panel A, except that MNase was omitted. FLAG-Akt was used as a non-specific control. Protein complexes were precipitated onto anti-FLAG or anti-c-MYC agarose. Input, 25% of input HA2-hEST1A. Blots were probed with anti-HA antibody, stripped, and reprobed with anti-FLAG antibody. Lower: as above, performed with hEST1A (a.a. 114-749), a fragment that exhibited non-specific interactions (lanes 16, 18-20). (D) Mutation of two DAT regions of hTERT does not affect interaction with hESTA(114-824). Experiment performed as in Panel C. Amino acid substitutions (NAAIRS) starting at amino acid 92 or 122, as indicated.

Although it was known that hEST1A interacts with hTERT *in vitro *independently of hTR [33], and that TPRs typically mediate protein-protein interactions [45], it was unknown whether the TPR was sufficient for the interaction between hEST1A and hTERT. Fragments of hEST1A spanning all or part of the TPR (502-824, 334-824, 114-749, 114-780) proved unsuitable for *in vitro *interaction analysis as they precipitated non-specifically (Figure 1C, and data not shown). An hEST1A fragment spanning a.a. 114-631 did not exhibit non-specific precipitation but failed to exhibit a significant interaction with hTERT fragments (Figure 1A, B). Increasing the C-terminal boundary to fully encompass the minimal TPR domain spanning a.a 695-761 (to a.a. 824) resulted in a reproducible enrichment of hEST1A(114-824) onto anti-FLAG agarose in the presence of FLAG-hTERT fragments spanning amino acids 1-200, 1-350, 201-560, 601-1132, but not fragments spanning 201-350 or 350-560, a control protein (Akt), or mock translation reactions containing no input cDNA (Figure 1A-C). The hTERT-hEST1A interactions were not perturbed by micrococcal nuclease, indicating that they did not require nucleic acids (Figure 1A, B). Taken together, these data identify at least one interaction interface within hTERT(1-350) that includes residues beyond a.a. 200 that are important (but insufficient in the context of a.a. 201-350) for an interaction with hEST1A(114-824).

To further refine the hTERT protein interaction interface within the hEST1A TPR, we introduced various mutations within a.a. 551-785 of hEST1A(114-824) that we predicted to lie on the protein surface (see below), and tested these fragments for an hTR-independent interaction with hTERT(1-350) or hTERT(601-1132) (Additional file 1, Figure S2). None of the 22 introduced mutations, including the 17 residues mutated between hEST1A a.a. 631 and 824, perturbed the interaction of hEST1A(114-824) with either hTERT fragment (data not shown) [51]. In addition, removal of a sequence in hEST1A (a.a. 708-724) corresponding to a putative disordered loop in hEST1C [46] did not disrupt the hEST1A-hTERT interaction (Figure 1C). Thus, despite the existence of a putative hTERT interaction interface in hEST1A (631-824) that includes a TPR domain (695-761), we were unable to identify residues within the TPR essential for this interaction.

We also tested the effect of mutations within the hTERT N-terminus on the interaction with hEST1A(114-824). Two DAT ("dissociates activities of telomerase") regions in hTERT, when substituted for six amino acids (NAAIRS) beginning at codons 92 or 122 [52-58], also proved dispensable for the interaction with hEST1A(114-824) (Figure 1D). DAT mutations have subtle effects on telomerase catalysis *in vitro *but dramatic deficits in telomere elongation and lifespan extension *in vivo *that can be partially rescued by fusion of hTERT-DAT to POT1 or TRF2 [52-58]. Our data suggest this *in vivo *deficit is not the result of a perturbed interaction with hEST1A.

### Non-conservation of EST1 TPR function between species

Recent data establish that hEST1C, of which the closest *S. cerevisiae *homolog is Ebs1, plays a similar role as Ebs1 in NMD [42]. Thus, we wished to determine if hEST1A or hEST1B might display functional similarity with ScEst1. Despite various strategies, we were unable to observe expression of full-length hEST1A, B, C in *S. cerevisiae*, which precluded analysis of whether human Est1 homologs might, alone or in combination, complement the senescence phenotype of strains lacking *EST1*. An alternate strategy was to test whether a region of Est1 containing the TPR could be "swapped" between species. Using an *in vivo *gap-repair cloning strategy in *S. cerevisiae *[59], the TPR of ScEst1 was replaced with the TPR of hEST1A, hEST1B or hEST1C or GFP(S65T) (the latter representing a similarly-sized domain as the TPR) (Additional file 1, Figure S3). The boundaries of the TPR were estimated according to the structure-based sequence alignment of EST1 homologs [32,33,46]. Specifically, the first nine alpha helices of hEST1C, or the corresponding region of hEST1A or hEST1B, were integrated into the corresponding region of ScEst1.

**Figure 2:**
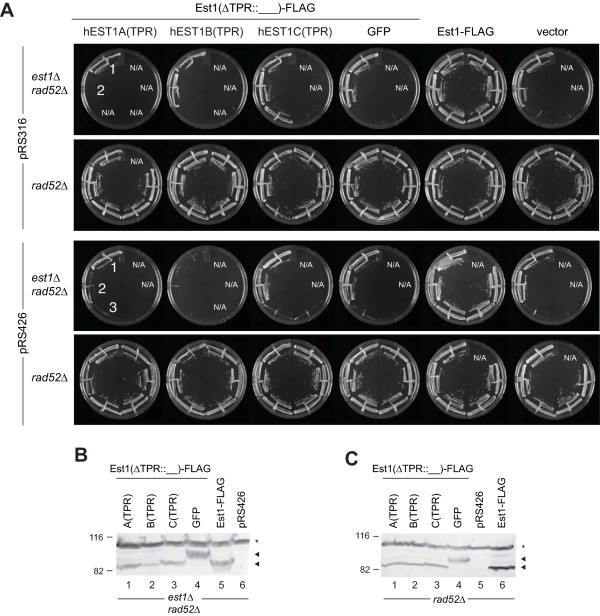
**Est1 hybrids do not rescue *est1Δ rad52Δ *strains or interfere with growth of *rad52Δ *strains**. (A) Heterozygous diploid *est1Δ rad52Δ *yeast were transformed with pRS316(*URA3*) or pRS426(*URA3*) plasmids alone ('vector') or plasmids expressing yeast/human EST1 hybrids in which the TPR of ScEst1 was replaced with the TPR of hEST1A/B/C, or GFP(S65T). Haploid spores were isolated and passaged every two days on plates containing synthetic dropout media lacking uracil. Summary plates of growth from the 1^st ^passage to 6^th ^passage (as shown, counter-clockwise) were prepared by re-streaking colonies from each passage onto one sector of a single plate (refer to Methods). Refer to Additional file 1, Figure S4 for summary of spore growth. (B, C) Yeast/human Est1 hybrid proteins were expressed. Western blot of cell lysates using anti-FLAG and anti-mouse IgG-HRP antibodies. Arrowheads indicate wild-type or hybrid Est1 proteins. A non-specific, cross-reacting band is indicated with an asterisk (*). Molecular mass (kDa) is indicated at the left.

**Figure 3:**
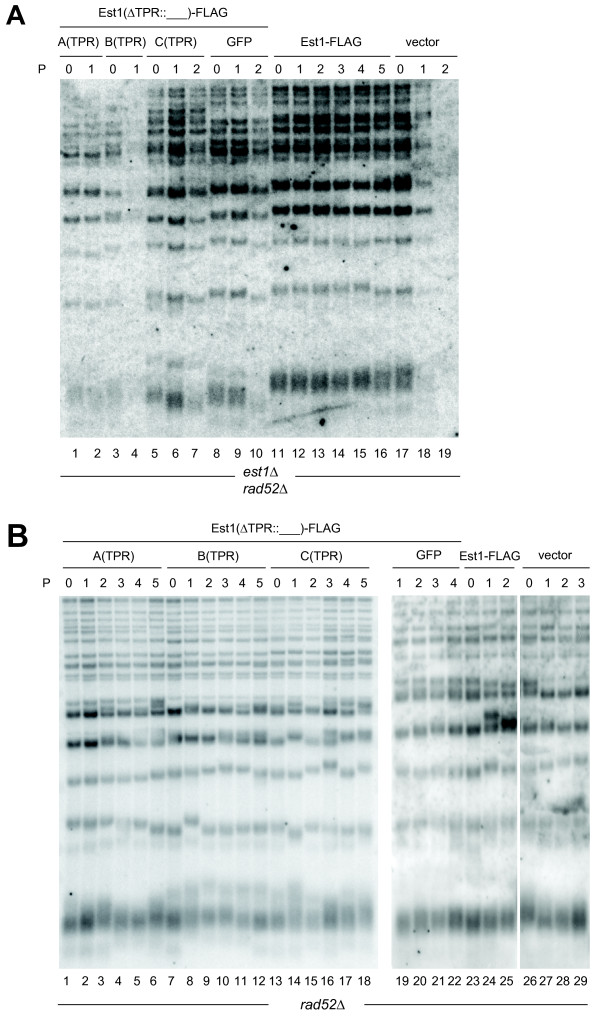
**Est1 hybrids do not maintain telomeres (*est1Δ rad52Δ*) or interfere with wild-type Est1 (*rad52Δ*)**. Telomere DNA Southern blots. Genomic DNA isolated from *est1Δ rad52Δ *(A) and *rad52Δ *(B) cells at the indicated passages (P; represented in Figure 2) was digested with XhoI. Strains expressed empty vector (pRS426; vector), wild-type Est1-FLAG, or Est1-FLAG hybrid proteins, as indicated. Telomeres were analyzed by Southern blotting using a (CACACCCA)_2_CC DNA probe. Lanes 1-18 and 19-28 represent different blots, and data between lanes 25-26 was omitted.

We first verified that *est1Δ *and *est1Δ rad52Δ *haploid strains generated from a diploid strain (S288C *EST1/est1Δ::NAT RAD52/rad52Δ::KAN*) underwent senescence as expected [1,8]. Next, we tested the ability of low-copy (pRS316) or high-copy (pRS426) hybrid Est1 expression plasmids (introduced into the *EST1/est1Δ RAD52/rad52Δ *strain prior to haploid selection) to rescue senescence in two separately isolated *est1Δ rad52Δ *haploid spores (Figure 2). Although the resultant TPR hybrid proteins were expressed (Figure 2B), neither the GFP(S65T)-Est1 hybrid nor any of the human TPR-Est1 hybrids proved sufficient to rescue senescence or maintain telomere length when spores were passaged every two days (Figure 2A, 3A, Additional file 1, Figure S4). Extension of the time between colony propagation from two days to four days, which promotes the rare survival (approximately 4-8%) of populations lacking telomerase and *RAD52 *[6], also failed to permit complementation with the Est1 TPR hybrid proteins (Additional file 1, Figure S4). The TPR hybrids also failed to exert a dominant interfering effect on cell viability or telomere length maintenance in an *EST1 rad52Δ *strain (Figure 2A, C, Figure 3B, Additional file 1, Figure S4). Therefore, replacement of the cognate TPR of ScEst1 with the TPR of hEST1A, B, or C neither complemented nor interfered with wild-type ScEst1.

### Dissection of Est1 TPR function by mutagenesis

**Figure 4:**
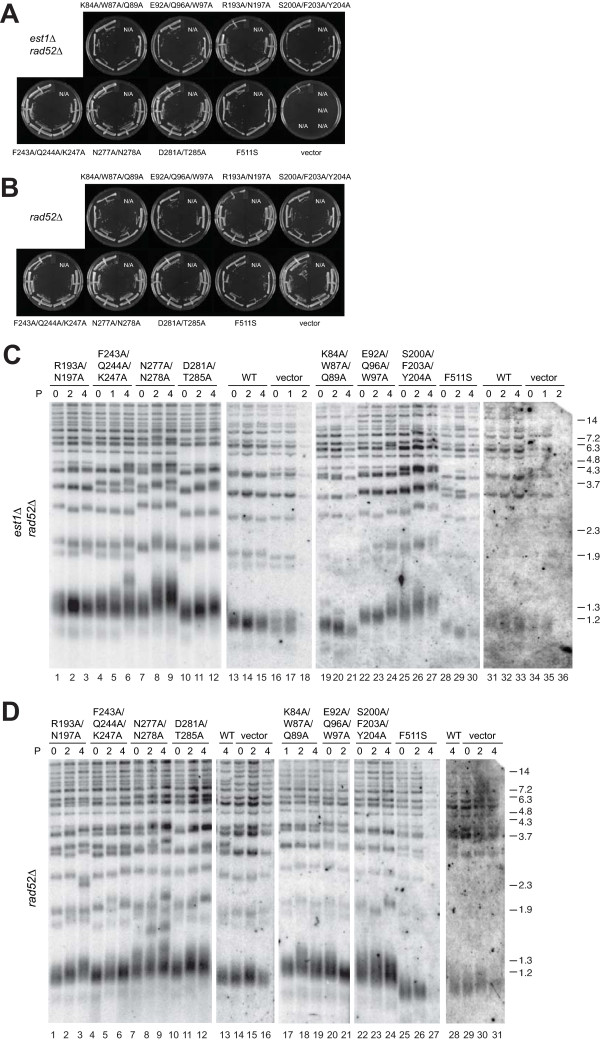
**Mutation of the Est1 TPR does not compromise viability in *S. cerevisiae *but alters telomere length homeostasis**. Heterozygous diploid *est1Δ rad52Δ *yeast were transformed with pRS426(*URA3*) plasmids expressing ScEst1-FLAG wild-type or indicated TPR mutants. Haploid *est1Δ rad52Δ *(A) and *rad52Δ *(B) spores were isolated and passaged every two days on plates containing synthetic dropout media lacking uracil. Refer to Additional file 1, Figure S5 for summary of spore growth. Telomere DNA Southern blots in haploid *est1Δ rad52Δ *(C) and *rad52Δ *(D) strains expressing empty vector (pRS426; vector), ScEst1-FLAG TPR mutants or wild-type Est1-FLAG, as indicated. Genomic DNA isolated from cells at the indicated passages (P; represented in A, B) was digested with XhoI. Telomeres were analyzed by Southern blotting using a (CACACCCA)_2_CC DNA probe. In (C), two blots are represented by lanes 1-18, and 19-36; in (D), two blots are represented by lanes 1-16, and 17-31. Gaps represent removal of redundant samples, or slight changes between contrast enhancement. DNA ladders are shown (kbp) at right.

**Figure 5:**
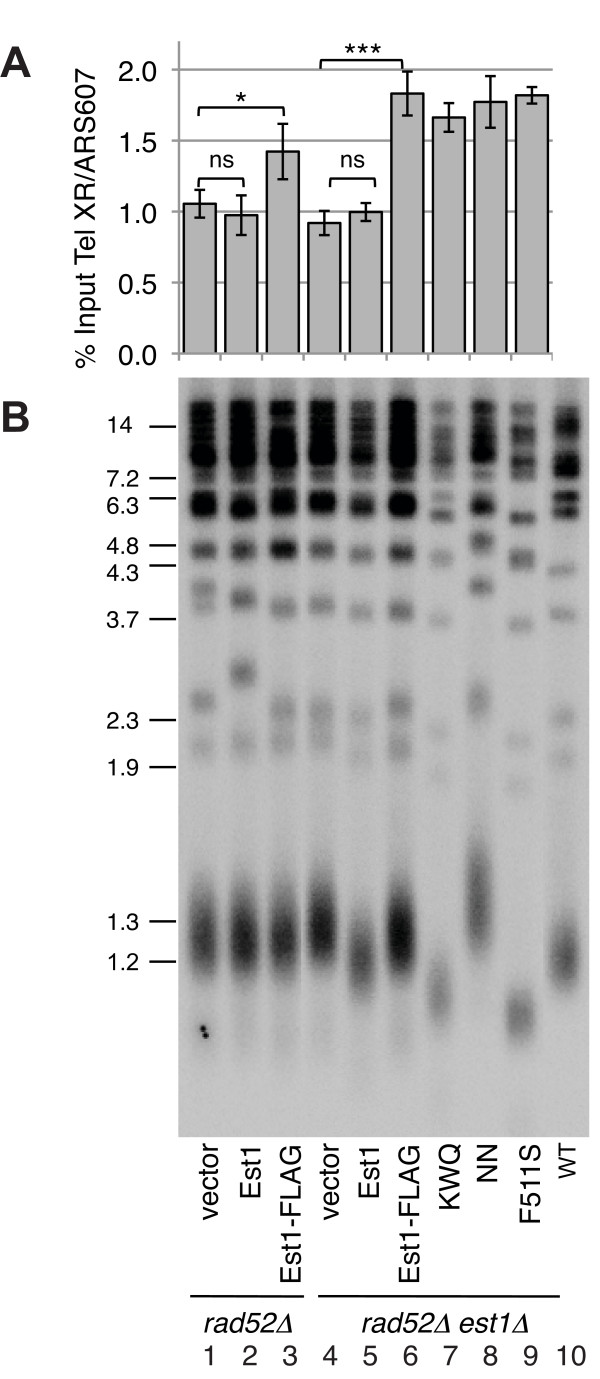
**Mutation of the Est1 TPR does not interfere with recruitment to telomeric chromatin**. Chromatin immunoprecipitation of FLAG-tagged Est1 proteins (in pRS426) was performed as described in Methods. Enrichment of telomeric DNA was determined by comparing the intensity of PCR products generated using primer pairs matching a subtelomeric locus on chromosome X (tel XR) or another single-copy genomic locus (ARS607) and normalising to the values corresponding to input DNA. Data represent mean ± S.D. for n = 4 independent samples. Significant differences relative to the vector control (indicated by asterisks; * p < 0.01, *** p < 0.0001) were determined by one-way ANOVA and the Tukey post-hoc test. Nomenclature: vector, pRS426 alone; Est1, untagged; Est1-FLAG; KWQ, Est1(K84A/W87A/Q89A)-FLAG; EQW, Est1(E92A/Q96A/W97A)-FLAG; NN, Est1(N277A/N278A)-FLAG. (B) TRF analysis of the same strains as in (A); lane 10, S288C (WT). DNA markers at left in kbp. Note that average telomere lengths are longer in r*ad52Δ *strains (lanes 1-3) compared with WT (lane 10), as expected [15,16]. All samples were resolved on the same gel, and irrelevant or blank lanes between lanes 3, 4 and 9, 10 were removed.

Since the TPRs of hEST1A, B, or C were unable to function in place of the TPR of ScEst1, we mutated residues within and just outside the ScEst1 TPR domain and assessed effects on function *in vivo*. Selected residues that we predicted to be exposed to the concave surface of the putative protein-protein interaction interface (according to the alignment of the ScEst1 sequence to the hEST1C TPR structure) were changed to alanine [46] (Additional file 1, Figure S2); all mutants complemented the viability of *est1Δ rad52Δ *strains over multiple generations and did not interfere with viability in *rad52Δ *strains (Figure 4A, B, Additional file 1, Figure S5). The average telomere length of cell populations expressing wild-type FLAG-tagged Est1 were compared with those expressing FLAG-tagged Est1 point mutants including Est1(F511S) - a mutant which confers viability in the absence of endogenous Est1 but maintains shorter-than-wild-type telomere lengths [19,60] (Figure 4C, lanes 13-15, 28-30). Like cells expressing Est1(F511S), cells expressing Est1(K84A/W87A/Q89A)-FLAG exhibited slightly shorter telomeres (Figure 4C, lanes 19-21, 31-33). Unexpectedly, *est1Δ rad52Δ *cells containing certain Est1-FLAG mutants - namely E92A/Q96A/W97A, R193A/N197A, S200A/F203A/Y204A, F243A/Q244A/K247A, N277A/N278A, or D281A/T285A - exhibited longer telomeres than cells containing wild-type Est1-FLAG (Figure 4C). Telomere lengths appeared to slightly increase upon successive passages with certain mutants such as N277A/N278A, E92A/Q96A/W97A and S200A/F203A/Y204A (Figure 4C, lanes 7-9, 22-24 or 35-36, and 25-27, respectively). The elongation phenotype was not due to overexpression of Est1, since telomere lengths in cells overexpressing wild-type Est1 (tagged or untagged) did not differ from strains lacking high-copy Est1 (Figure 5B). The differences in telomere lengths relative to wild-type Est1 and to each other were reproducible with different strain isolates (data not shown). Although we did not test the protein expression levels of all point mutants, larger perturbations in Est1 (e.g., the TPR 'swaps') did not alter Est1 levels relative to wild-type Est1-FLAG (Figure 2B). Thus, the different effects of TPR mutations on telomere length suggest that distinct residues in the vicinity or within the TPR domain function in telomere length maintenance, while other residues may function to limit telomere elongation.

The Est1-FLAG mutants were also tested for their effect on telomere length homeostasis in an *EST1 rad52Δ *strain (Figure 4D, Additional file 1, Figure S5). Est1(K84A/W87A/Q89A), which elicited shorter telomeres in an *est1Δ rad52Δ *background, did not elicit telomere attrition in the presence of wild-type Est1 (Figure 4D, lanes 17-19, 30-31). Expression of the Est1-FLAG mutants (R193A/N197A, F243A/Q244A/K247A, N277A/N278A, D281A/T285A, E92A/Q96A/W97A, or S200A/F203A/Y204A) in a *rad52Δ *background resulted in slightly longer telomeres that persisted for five passages (Figure 4D). Thus, these mutants may interfere with an ability of wild-type Est1 to inhibit telomere elongation. Est1(F511S) led to shorter telomeres in a *rad52Δ *background similar to the effect observed in a *rad52Δ est1Δ *strain (Figure 4D, lanes 25-27), suggesting an ability of this mutation to interfere with wtEst1 function. The FLAG tag itself did not interfere with Est1 function, since telomeres in strains overexpressing Est1-FLAG (in either a *rad52Δ *or an *est1Δ rad52Δ *background) or untagged Est1 were maintained at a similar length as strains expressing only endogenous Est1 (Figure 4D, compare lane 13 with lanes 14-16 and Figure 4C, lanes 13-15, Figure 5B). Collectively, these data suggest that mutations within the Est1 TPR permitted complementation of *est1Δ *cells; in some instances, concomitant telomere shortening or elongation was observed that suggested both positive and negative regulatory roles for the TPR in telomere length homeostasis.

### Recruitment of Est1 point mutants to telomeric chromatin

To test whether mutation of the Est1 TPR affected recruitment to the telomere, we performed chromatin immunoprecipitations (ChIP) experiments. Yeast lysates were subjected to crosslinking and sonication to obtain chromatin fragments approximately 500 bp in length, followed by immunoprecipitation onto anti-FLAG M2 resin. PCR analysis was performed using primers designed to amplify a single-copy genomic locus (ARS607) or the subtelomeric region of the right arm of chromosome X (tel XR). After normalization to both input genomic DNA and the ARS607 amplification signal, wild-type Est1-FLAG (Figure 5A, lanes 3, 6) exhibited a significant fold-enrichment at telomeric chromatin relative to same strain lacking ScEst1-FLAG (Figure 5A, lanes 1, 4). We next tested three FLAG-tagged Est1 mutants (K84A/W87A/Q89A, N277A/N278A, F511S) for enrichment at telomeric chromatin in the *est1Δ rad52Δ *background. All three mutants exhibited telomeric enrichment similar to Est1-FLAG (Figure 5A). Notably, mutants associated with telomere elongation (N277A/N278A) or shortening (F511S, K84A/W87A/Q89A) exhibited equivalent enrichment. The similar levels of enrichment also suggest that fluctuations in telomere length are not simply due to changes in the level of Est1 protein recruited to telomeric heterohromatin (Figure 5B). These data suggest that the effects on telomere length equilibrium conferred by the Est1 TPR mutants may involve steps downstream of recruitment to the telomere.

## Discussion

### Species-specific differences in the TERT-EST1 interaction

Our observation that N- and C-terminal regions of hTERT can interact with hEST1A (114-824) independently of hTR extends previous findings [33,47]. Redon *et al*. showed that an hTR-dependent interaction occurs between hTERT(147-311) and hEST1A(212-381), and that hTERT(147-311) may interact with hEST1A fragments beyond a.a. 212-502 independently of hTR [47] (Additional file 1, Figure S6). Collectively, our results suggest that hTERT forms protein-protein contacts with the TPR of hEST1A. As yet, the specific amino acids responsible for these interactions have not been identified. That the hEST1A TPR exhibits multiple contacts with discrete hTERT fragments is not without precedent; the hTERT amino-terminus (hTEN) also exhibits specific interactions with itself and the hTERT C-terminus, suggesting that an hEST1A interaction could be bridged by intramolecular hTERT interactions *in vivo *[56,61-63]. In addition, hTEN exhibits specific interactions with telomeric DNA and TPP1 that stimulate repeat addition processivity [64-68]. Taken together, these data underscore that multiple protein-protein and protein-DNA interaction interfaces serve to regulate hTERT function *in vivo*.

Like the interaction between hEST1A and hTERT, the interaction between Est1 and Est2 may involve both protein-protein and protein-RNA contacts. Est1 and Est2 interact with separate regions of Tlc1 [26-28,69], and Est1 does not co-purify with Est2 in a *tlc1Δ *strain, suggesting that Tlc1 is required to assemble the telomerase complex [60]. However, recent findings suggest that recombinant ScEst1 stimulates telomerase activity even when the Tlc1-Est1 interaction is compromised, and that Tlc1 stimulates but is not required for an Est1-Est2 interaction in RRL [70]. These results suggest that Est1 and telomerase may also interact through protein contacts.

The TPR of ScEst1 may be involved in binding Tlc1. Mutations at residues that we predicted to reside on the convex surface of the domain (118/122/123 and 222/223/226) elicit telomere shortening and perturb association with Tlc1 [60]. None of the mutations that we introduced into the putative concave surface of the TPR of hEST1A reduced the interaction with hTERT. Mutagenesis of the convex surface of hEST1A TPR may yet identify an hTERT interaction interface.

The TPRs within hEST1C(1-497) and hEST1A(545-785) bind a phosphopeptide within UPF1; these interactions are reduced by K66E/R163E and R625E/R706E mutations (on the concave surface), respectively, or by phosphatase treatment [46]. In our studies, the R625E/R706E or D703/R706/Y707/Y724 mutations in hEST1A(114-824) did not disrupt the interactions with hTERT(1-350) or (601-1132). Since hTERT is phosphorylated by Akt and c-Abl *in vivo *[71,72], hEST1A may bind an as yet unmapped phosphomotif in hTERT. Alternatively, UPF1 and hTERT may interact with distinct interfaces of the hEST1A TPR. By analogy, in *S. cerevisiae*, components of the anaphase-promoting complex (Cdc16, Cdc23, and Cdc27) each contain multiple TPR repeats. Mutation of one TPR within Cdc27 impairs its interaction with Cdc23, but does not appreciably affect binding to Cdc16, or Cdc27 self-association [73].

### A role for the ScEst1 TPR in curtailing telomere elongation downstream of telomere binding

We found that mutations within the ScEst1 TPR that affected telomere length equilibrium did not reduce the association of Est1 with telomeric heterochromatin in asynchronous cultures. Since Est1 recruitment to telomeres peaks in S phase [17,18,20], further experiments in synchronized cultures may reveal further nuances of the influence of these mutations on the temporal regulation of telomere recruitment. The fact that the mutants showed a comparable enrichment to that of wild-type Est1 suggests that these ScEst1 TPR residues are nonetheless dispensable for an interaction with Cdc13, and is consistent with the notion that the perturbation of telomere length reflects a defect downstream of telomere recruitment.

While certain mutations predicted to disrupt the concave surface of the Est1 TPR elicited telomere shortening, other similarly predicted mutations elicited telomere elongation. The concave surface of the Est1 TPR may negatively regulate telomere length through an interaction with Ies3 - a component of the INO80 chromatin remodeling complex [74]. As demonstrated by yeast two-hybrid and co-immunoprecipitation experiments, Est1 and Ies3 interact, and deletion of Ies3 results in telomere lengthening [74]. Mutation of three amino acids within the TPR of Est1 (G199A/P236A/N278A) abolishes the Ies3 interaction [74]. This triple mutation impairs Tlc1 binding and elicits telomere shortening, thus precluding a direct assessment of the Ies3-Est1 interaction in telomere length regulation [74]. In contrast, we observed that alanine replacement of N277/N278 in Est1 led to telomere elongation. Thus, it is possible that the N277A/N278A mutation might disrupt interaction with Ies3 without perturbing Tlc1 association, which would support a direct role for the Ies3-Est1 interaction in the negative regulation of telomere length by the INO80 complex [74]. Our results indicate that the TPR-containing region within ScEst1 positively and negatively regulates telomere length, and that the concave surface of the TPR domain appears important for Est1 function.

### Functional specialization of key cellular processes within and between species

We observed that the TPRs of human EST1 proteins could not substitute for the ScEst1 TPR. Functional differences in other telomere-associated proteins have been noted across species. Two related 5'-3' helicases in *S. cerevisiae*, Pif1 and Rrm3, perform non-overlapping functions in inhibiting telomerase recruitment and promoting DNA fork progression, respectively [75], and both contribute to mitochondrial DNA stability in distinct ways [76,77]. In *S. pombe*, the single Pif1/Rrm3 ortholog Pfh1 is essential; some, but not all, of its diverse roles in mitochondrial, nuclear and telomere DNA replication are complemented by ScRrm3 [78-81]. In mammals, although the Pif1/Rrm3 ortholog interacts with telomerase, it is dispensable for viability, telomere length maintenance, and chromosome healing [82-84]. Similarly, Rif1 plays a critical regulatory role in telomere homeostasis in yeasts [reviewed in [14,85-89], while in mammals Rif1 modulates genome stability in response to DNA damage and appears dispensable for telomere length regulation [90-93].

In *S. cerevisiae*, the NMD-related function of EST1 homologs may have been delegated primarily to Ebs1, which shares the most sequence similarity (particularly in the TPR) with hEST1C [42]. Strains lacking *EBS1 *possess short telomeres [26] and, unlike *est1Δ *strains, exhibit a defect in NMD [37,42]. Over-expression of hEST1C or Ebs1 in *S. cerevisiae *disrupts NMD [42]. In *A. thaliana*, EST1C/SMG7 is an essential gene with a role in NMD and meiotic cell cycle progression, but possesses no apparent role in telomere integrity or length regulation [94]. It will be interesting to determine whether mutations exist within the hEST1A TPR that selectively affect NMD or an interaction with hTERT. If a telomerase-specific interaction interface within hEST1A exists, it may represent an attractive target for telomerase inhibition in cancer since its short-term perturbation might spare other essential functions of EST1 related to telomere end protection or NMD.

## Conclusions

A fragment of hEST1A of approximately 700 a.a. and encompassing the tetratricopeptide repeat (TPR, a.a. 695-761) was sufficient for nucleic acid-independent interactions with the N-terminus of hTERT (a.a. 1-350). The TPR of ScEst1 could not be complemented genetically by TPRs of the human Est1 homologs hEST1A, hEST1B, or hEST1C, suggesting that the former has evolved species-specific interactions that cannot be substituted by human EST1 TPRs. Mutagenesis within and just outside the ScEst1 TPR identified residues important for telomere length homeostasis but not recruitment to telomeric heterochromatin. Collectively, these data identify species-specific roles of the Est1 TPR domain in the recognition of hTERT and regulation of telomere length.

## Methods

### Co-immunoprecipitation of hTERT and hEST1A fragments and micrococcal nuclease treatment

pCR3-FLAG-hTERT-FLAG and pcDNA3.1-HA2-hEST1A constructs were expressed in rabbit reticulocyte lysates (RRL) according to the manufacturer's instructions (Promega Corp.) in the absence of hTR. The inclusion of hTR RNA in hTERT translation reactions did not affect interactions with hEST1A (data not shown). Mock reactions contained no cDNA. Reactions were incubated separately for 90 min at 30°C. Fifteen to 20 μL of each reaction was mixed in a total volume of 500 μL with cold CHAPS buffer (0.5% w/v CHAPS, 10 mM Tris-Cl pH 7.5, 10% v/v glycerol, 100 mM NaCl, 1 mM MgCl_2 _1 mM DTT, Roche EDTA-free Complete protease inhibitor cocktail) and agitated gently for 1 hr at 4°C. FLAG-tagged complexes were immunoprecipitated with pre-equilibrated anti-FLAG M2 agarose resin (Sigma). Anti-c-Myc agarose (BD Biosciences) was employed as a negative control resin. For micrococcal nuclease digestions, immunoprecipitates were washed twice with cold CHAPS buffer and treated with 40 U/μL micrococcal nuclease (New England Biolabs) at 30°C for 45 min in buffer containing 50 mM Tris-Cl pH 7.9, 5 mM CaCl_2 _and 1 mg/mL bovine serum albumin, or mock-treated in buffer without micrococcal nuclease. Reactions were terminated by the addition of excess EGTA, and immunoprecipitates were washed twice in cold CHAPS buffer. For experiments not involving micrococcal nuclease, immunoprecipitates were washed three times in cold CHAPS buffer.

Immunoprecipitates were boiled in SDS-PAGE loading dye. Proteins were resolved through 4-20% w/v Tris-Glycine Novex gels (Invitrogen), transferred to PVDF membranes (Invitrogen), and subjected to immunoblotting with anti-HA-HRP (3F10; Roche). Where indicated, antibodies were removed by incubation in stripping buffer (100 mM β-mercaptoethanol, 62.5 mM Tris-Cl pH 6.8, 2% w/v SDS) for 10 min at 50°C. Membranes were washed in 1X TBS containing 0.05% v/v tween20 (BioRad), and re-blotted with mouse anti-FLAG M2 (Sigma) and sheep anti-mouse IgG-HRP (GE Healthcare), or anti-hTERT(RT domain) [95] and donkey anti-rabbit IgG-HRP (GE Healthcare). Blots were developed with ECL Plus (GE Healthcare) and exposed to film (Kodak) or captured using a Typhoon Trio variable mode imager.

In Figure 1B, immunoprecipitation data from four independent replicates of the experiment shown in Figure 1A were quantified as follows. Firstly, the raw intensity of each hEST1A fragment (114-824 or 114-631) immunoprecipitated onto anti-FLAG resin was divided by the average intensity (n = 4) of the fragment in the mock immunoprecipitation lane (no added TERT). Secondly, the raw intensity of each hTERT fragment precipitated onto anti-FLAG resin was expressed as a ratio relative to the average intensity value of the same hTERT fragment (n = 4). The normalized hEST1A value was divided by the normalized hTERT fragment value in each experiment, and the mean and standard deviation of these ratios is illustrated in Figure 1B. Statistical significance was determined by ANOVA analysis of the normalized ratios, assuming non-paired samples, and applying the Tukey post-hoc test for pairwise comparisons where confidence levels exceeded 95%.

### Generation of yeast strains

The diploid S288C *est1Δ::KAN/EST1 *strain (*MAT*a*/α his3Δ ura3Δ leu2Δ met15Δ LYS2*^*+ *^*TRP1*^*+*^) was a gift from Brenda Andrews (University of Toronto). The diploid was sporulated to obtain a haploid S288C *est1Δ::KAN *strain (*MAT*a). The *KAN *gene replacement cassette was substituted with a *NAT *cassette by transformation and appropriate selection to obtain S288C *est1Δ::NAT*. This strain was crossed with a haploid S288C strain (*MATα*) of the same genetic background to create a diploid S288C *est1Δ::NAT/EST1 *strain. *RAD52 *was deleted by transformation with a *KAN *gene replacement cassette followed by appropriate selection to obtain S288C *est1Δ::NAT/EST1 rad52Δ::KAN/RAD52*. Gene deletions were verified by PCR and restriction enzyme digestion.

### Yeast expression vectors

Open reading frames for hEST1A/SMG6 [GenBank:NM_001170957; hereafter referred to as hEST1A], hEST1B/SMG5 [GenBank:AY168922; hEST1B] and hEST1C/SMG7 variant 2 [GenBank:NM_201568; hEST1C] were cloned into low-copy (1-2 copies per cell) pRS316 (*CEN6*, *ARS4, URA3*) and high-copy (~50 copies per cell) pRS426 (*2 μ ori, REP3, FRT, URA3*) expression plasmids [96,97]. pRS316 and pRS426 plasmids were obtained from Raymund Wellinger (University of Sherbrooke).

### Generation of yeast/human EST1 TPR hybrids

The boundaries of the TPR were selected according to the structure-based alignment of EST1 sequences [32,33,46] (Additional file 1, Figure S3A). Plasmids encoding hybrid EST1 proteins in which the TPR of ScEst1 was replaced with the TPR of hEST1A, hEST1B, or hEST1C were generated by the *in vivo *gap-repair cloning method [59]. In the first step, pRS316(*URA3*)-Est1-FLAG was digested with NruI and PflMI which cleaved the TPR coding sequence at unique restriction sites in the plasmid (Additional file 1, Figure S3B). In parallel, the sequence encoding the TPR of hEST1 was amplified by PCR using hybrid primers. The 5' ends of the forward primers contained 45 nt of sequence identical to sense strand immediately upstream of ScEst1 codon 14 (the TPR sequence starts at codon 14). The 3' ends of the primers contained 30 nt of sequence identical to the sense strand of the TPR coding sequence of hEST1A (starting at codon 551), hEST1B (codon 25), or hEST1C (codon 2). GFP(S65T) (amino acids 1-237) was also engineered in place of the TPR of ScEst1 as a control for disruption of the domain. The forward primers were as follows: Est1p13/hEST1A551, 5'-(PO_4_)-GGCTTAATGGATAATGAAGAAGTTAACGAAGAATGTATGAGATTACACAGGCTTCTCCGGGTGGCTGACAACCAG-3'; Est1p13/hEST1B25, 5'-(PO_4_)-GGCTTAATGGATAATGAAGAAGTTAACGAAGAATGTATGAGATTACGGGCTGTGGTGGAGGCTGTGCATCGACTT-3'; Est1p13/hEST1C2, 5'-(PO_4_)-GGCTTAATGGATAATGAAGAAGTTAACGAAGAATGTATGAGATTAAGCCTGCAGAGCGCGCAGTACCTCCGGCAG-3'; Est1p13/GFP, 5'-(PO_4_)-GGCTTAATGGATAATGAAGAAGTTAACGAAGAATGTATGAGATTAAGTAAAGGAGAAGAACTTTTCACTGGAGTT-3'. The 5' termini of the reverse primers contained 45 nt of sequence identical to the antisense strand of ScEst1 immediately downstream of codon 289 (the TPR sequence ends at codon 289). The 3' termini of the primers contained 30 nt of sequence identical to the antisense strand at the end of the TPR encoding sequence of hEST1A (upstream of, and including codon 785), hEST1B (codon 262), or hEST1C (codon 236). The reverse primers employed were as follows: Est1p290/hEST1A785, 5'-(PO_4_)-TTTTGACACAAGAATTGCCAATTTTTTCATCAGACGTCTTCTTTCCTTCCGCTTGGTCTCTTCAAACAAGCTCAT-3'; Est1p290/hEST1B262, 5'-(PO_4_)-TTTTGACACAAGAATTGCCAATTTTTTCATCAGACGTCTTCTTTCCATTTTGGCTGCCTTGTCATACAGCCGCTT-3'; Est1p290/hEST1C236, 5'-(PO_4_)-TTTTGACACAAGAATTGCCAATTTTTTCATCAGACGTCTTCTTTCGCTTTCCAGTGCTTTAGAAAGTGCTTTTTG-3'; Est1p290/GFP, 5'-(PO_4_)-TTTTGACACAAGAATTGCCAATTTTTTCATCAGACGTCTTCTTTCTTTGTATAGTTCATCCATGCCATGTGTAAT-3'. Primers were obtained from Invitrogen. The gel-purified linearized plasmid DNA and PCR products were transformed into *S. cerevisiae *(haploid S288C *est1Δ::NAT RAD52*) and grown on solid SD media containing clonNAT (nourseothricin) (Werner BioAgents, Germany) to select for the *est1Δ *genotype. The media also lacked uracil in order to select for gap repair of the linearized Est1 plasmid. The resultant plasmids were recovered using the Zymoprep II Yeast Plasmid Miniprep kit (Zymo Research) and amplified in *E. coli *via transformation and maxiprep (Qiagen). The coding sequences of the hybrid genes, including the junctions between human and yeast EST1 sequences, were verified by DNA sequencing. To create a set of pRS426 vectors, NotI/SalI fragments from the pRS316 vectors were ligated into the NotI/SalI sites of pRS426. The following hybrid genes were constructed: Est1(1-13)/hEST1A(551-785)/Est1(290-699)-FLAG; Est1(1-13)/hEST1B(25-262)/Est1(290-699)-FLAG; Est1(1-13)/hEST1C(2-236)/Est1(290-699)-FLAG; and Est1(1-13)/GFP(S65T)/Est1(290-699)-FLAG.

### Manipulation and propagation of yeast on solid media

Diploid yeast cultures were transformed according to a high-efficiency transformation protocol [98]. Diploids were sporulated on solid media, asci were digested with zymolyase, and haploid spores were dissected under a microscope using a microneedle (Singer, UK). Haploid spores of the desired genotype were streaked onto plates containing synthetic dropout media lacking uracil (SD-URA) to select for maintenance of plasmids expressing the *URA3 *gene. After two or four days of growth at 30°C (P1, passage 1 or approximately 20 generations), a single colony was re-streaked onto a second plate and incubated as described above (P2). Meanwhile, the P1 plate was stored at 4°C. This scheme was continued until P4 or P5 plates were obtained. To demonstrate growth from P1-6, one colony from each "passage" plate stored at 4°C (including the starting haploid spore from the tetrad dissection plate) was re-streaked onto a sector of a "summary plate" which was then incubated at 30°C for a final two or four days. Plates were imaged on a Bio-Rad Molecular Imager Gel Doc XR System under white light. Images were captured below the point of signal saturation.

### Yeast protein extraction and western blotting

Protein extracts were prepared as described [98]. Proteins were boiled in SDS-PAGE buffer, resolved through 5% w/v 29:1 acrylamide:bisacrylamide gels, transferred to PVDF membranes, and subjected to western blotting using mouse anti-FLAG M2 antibody (Sigma). Sheep anti-mouse IgG-HRP (GE Healthcare) was employed as secondary antibody. Blots were developed with ECL Plus reagents, and fluorescent signals were captured using a Typhoon Trio variable mode imager (GE Healthcare).

### Telomere length analysis by Southern blot

A single colony growing on solid media was inoculated into 5 mL SD-URA and grown overnight at 26°C. Cells were pelleted and genomic DNA was extracted and purified as described [99]. Genomic DNA (5 μg) was digested with XhoI overnight at 37°C. Restriction fragments were resolved through 0.7% w/v agarose gels at 45 V (2 V/cm). DNA was denatured in-gel by incubation with 0.5 M NaOH and 1.5 mM NaCl, and neutralized in buffer containing 1.5 M NaCl and 0.5 mM Tris-Cl pH 7.5. DNA was transferred to Hybond-N+ membranes in 20X SSC. Following transfer, membranes were rinsed in 2X SSC. Telomeric DNA was hybridized to a ^32^P 5'-end-labeled (CACACCCA)_2_CC probe in Church buffer (0.5 M NaPO_4 _pH 7.2, 1% w/v BSA, 7% w/v SDS, 1 mM EDTA), then washed with 1X SSC/0.1% w/v SDS. Membranes were exposed to a phosphorimager screen and scanned using a Typhoon Trio variable mode imager.

### Mutagenesis of hEST1A and ScEst1 TPRs

The structure-based sequence alignment of the TPR-like/TPR of hEST1C/SMG7 and EST1 homologs [32,33,46] was used as a guide to select residues in hEST1A(551-785) and ScEst1(14-289) that may be surface-exposed. The design of PCR primers for site-directed mutagenesis was aided by The Primer Generator [100] or SiteFind [101] and primers were obtained from IDT or Operon. The incorporation of the intended mutations into the respective cDNAs (and the absence of unwanted mutations) was confirmed by DNA sequencing.

### Chromatin immunoprecipitation

Yeast cells were grown to logarithmic phase in liquid culture to an optical density (O.D. 600 nm) of 1.0. Fifty mL of each culture was adjusted to 1% v/v formaldehyde (Sigma) and incubated at room temperature for 45 min. The crosslinking reaction was terminated by addition of glycine to a final concentration of 135 mM. Samples were washed three times in ice cold PBS at 4°C, and resuspended in 500 μL FA buffer (50 mM Hepes pH 7.6, 140 mM NaCl, 1 mM EDTA, 1% v/v Triton X-100, 0.1% v/v Na-deoxycholate, Roche EDTA-free Complete protease inhibitor cocktail) per 50 O.D._600 _cells. Samples were sonicated for four cycles of 10 s followed by 30 s on ice, using a 3.2 mm microtip (VWR). Sonication resulted in fragmentation of chromatin to an average size of 500 bp (range 300-800 bp) as determined by agarose gel electrophoresis. Two hundred μL sonicated lysate was incubated overnight at 4°C with anti-FLAG-M2 agarose resin (Sigma). After extensive washing with FA buffer, complexes were eluted from beads using 300 μg/mL FLAG peptide (Sigma). Cross-linked DNA was released by the addition of reverse cross-linking buffer (10 mM Tris pH 8, 4 mM EDTA, 100 mM NaCl) and incubation at 65°C for 16 hr. Samples were then incubated for 3 hr at 56°C after the addition of Proteinase K (0.3 mg/mL) and glycogen (0.5 mg/mL). DNA was extracted and PCR amplification was performed on approximately 10 ng template DNA using primers specific to a unique subtelomeric sequence of chromosome XR (XR F: 5'-TACCCTGTCCCATTTCATTATACC-3', XR R: 5'-TACAAGTGCAAGTGAGTATGGCAT-3') or to an internal non-telomeric region at ARS607 (ARS607 F: 5'-GTGGTGATATAAACACTACATTCGC-3', ARS607 R: 5'-GCTTTCTAGTACCTACTGTGCCG-3'). PCR products were separated on a 6% w/v polyacrylamide gel in 1X TBE. The relative intensity of the subtelomere-specific PCR product was calculated using ImageQuant software (GE Healthcare). A region of interest containing each PCR product was defined, and the local average background signal was subtracted. The resultant subtelomere-specific values were then divided by a value for the corresponding internal loading control (ARS607), and expressed as a percentage of the signal generated from input DNA. In Figure 5, the ANOVA analysis was performed on sample values normalized to the values for the vector control strain within each experiment to enable between-experiment comparisons. By varying the concentration of DNA (in separate experiments), PCR reactions for ChIP samples and input DNA were determined to be in the linear range of amplification. Statistical analysis was carried out using GraphPad Prism 5.0, employing one-way ANOVA and Tukey's post-hoc test.

## List of abbreviations

CHAPS: 3-(3-cholamidopropyl)dimethylammonio-1-propanesulfonate; ChIP: chromatin immunoprecipitation; EST: ever shorter telomeres; NMD: nonsense-mediated mRNA decay; P: passage; PIN: PilT N-terminal domain; PTC: premature termination codon; TERT: telomerase reverse transcriptase; TR: human telomerase RNA; Tlc1: telomerase RNA; Sc: *Saccharomyces cerevisiae*; TBS: Tris-buffered saline; TPR: tetratricopeptide repeat; SDS: sodium dodecyl sulphate.

## Authors' contributions

DCFS, CL and LH designed the experiments. ADK, DCFS, CL and FP performed the experiments. All authors analyzed the data. DCFS and LH wrote the manuscript. All authors read and approved the final manuscript.

## Supplementary Material

Additional file 1 **Supplementary Figures**. Figure S1. Schematic representation of human and *S. cerevisiae *EST1 homologs. Figure S2: Comparative sequence analysis of EST1 at positions selected for mutation. Figure S3: Schematic representation of yeast/human Est1 hybrid proteins. Figure S4: Schematic representation of colony growth in Figure 2. Figure S5: Schematic representation of colony growth in Figure 4. Figure S6: Summary of hTERT/hEST1A co-IP experiments. Click here for file
