# Supplementary material for: The TPR-containing domain within Est1 homologs exhibits species-specific roles in telomerase interaction and telomere length homeostasis
Source: BMC Mol Biol. 2011 Oct 18;12:45. doi: 10.1186/1471-2199-12-45 (PMC3215184; doi:10.1186/1471-2199-12-45)
Supplement: Additional file 1 — Supplementary Figures. Figure S1. Schematic representation of human and S. cerevisiae EST1 homologs. Figure S2: Comparative sequence analysis of EST1 at positions selected for mutation. Figure S3: Schematic representation of yeast/human Est1 hybrid proteins. Figure S4: Schematic representation of colony growth in Figure 2. Figure S5: Schematic representation of colony growth in Figure 4. Figure S6: Summary of hTERT/hEST1A co-IP experiments. [file 1471-2199-12-45-S1.PDF]

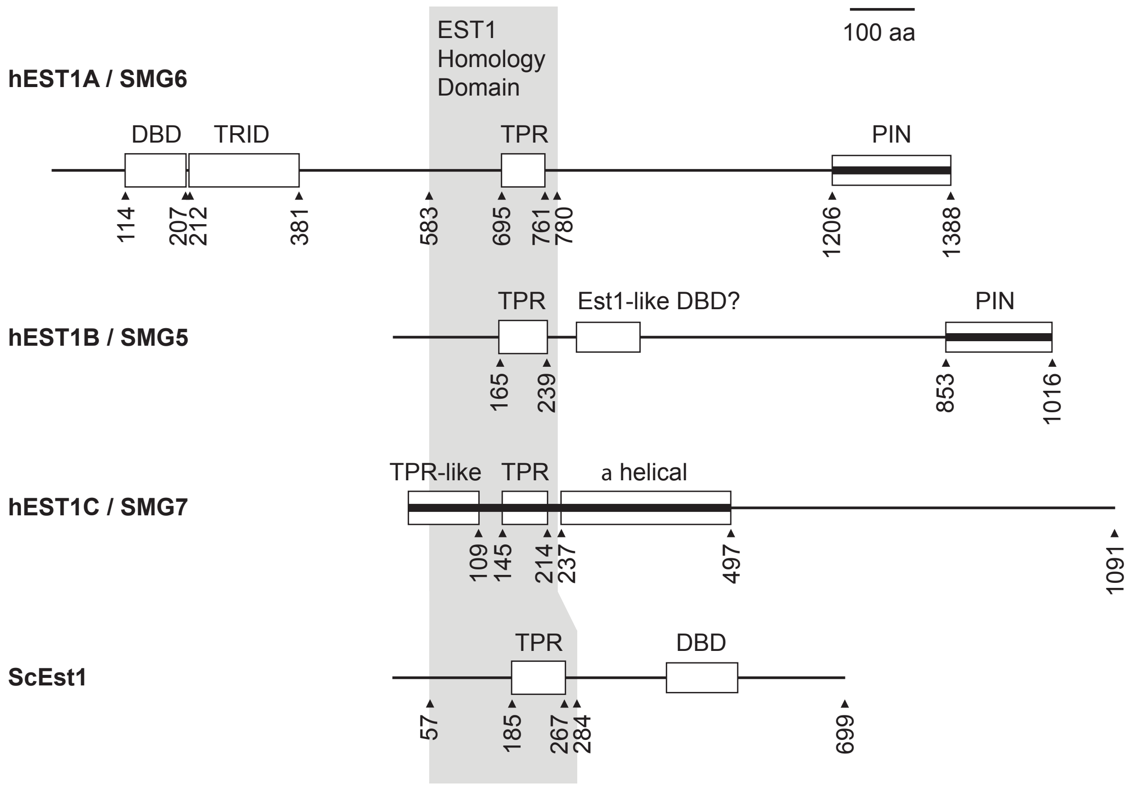

Figure S1: Schematic representation of human and *S. cerevisiae* EST1 homologs.
The region of highest homology among EST1 proteins (EST1 homology domain) is indicated by the shaded area. In this region, TPR domains are common to EST1 proteins. White boxes indicate featured domains. (hEST1A / SMG6) Amino acid numbers and domains correspond to isoform 2 [GenBank:NP_001164428.1]. DBD, N-terminal DNA binding domain [33; Cruickshank and Harrington, unpublished]. TRID, hTR-interaction domain [47]. PIN, nuclease domain [PDB:2HWW] [49]. (hEST1B / SMG5) [GenBank:AAO17582]. Est1-like DBD resembles the DNA binding domain (DBD) of Est1 [48]. PIN, nuclease domain [PDB:2HWY] [49]. (hEST1C / SMG7) Splice variant 2 is depicted [Genbank:NM_201568]. TPR-like and TPR domains of hEST1C assume a TPR-fold architecture upstream of an alpha-helical domain [PDB:1YAO] [46]. Structural data are available for regions represented by thick, dark lines.

hEST1A mutants				hEST1A[a]	hEST1B[b]	hEST1C[c]	ScEst1	Sc mutants
			L	551 H	25 R	2 S	14 F	
			E	552 R	26 A	3 L	15 F	
			M	555 R	29 E	6 A	18 A	
			A	558 D	32 H	9 L	21 H	
			D	559 N	33 R	10 R	22 L	
			M	561 E	35 D	12 A	24 K	
			A	565 S	39 C	16 K	28 S	
			D	566 N	40 N	17 A	29 R	
			N	569 S	43 A	20 T	32 C	
			K	593 E	63 C	40 Y	56 E	
			N	604 D	74 Y	52 L	78 V	
			V	605 N	75 G	53 D	79 I	
			E	606 Q	76 R	54 K	80 P	
		D	D	607 N	77 K	55 K	81 L	
		L		610 Q	80 E	58 Q	84 K	A
				613 W	83 W	61 W	87 W	A
			E	614 K	84 R	62 N	88 L	
			D	615 N	85 K	63 H	89 Q	A
			F	618 Y	88 Y	66 K	92 E	A
			L	619 Q	89 E	67 N	93 P	
			V	622 E	92 Q	70 T	96 Q	A
				623 K	93 L	71 T	97 W	A
E	E	E	E	625 R	95 K	73 Q	99 E	
		E	E	626 Q	96 T	74 G	100 H	
		E		629 K	99 K	77 K	103 H	
			M	688 K	165 D	145 S	185 F	
			E	696 R	173 R	153 H	193 R	A
				700 C	177 Y	157 H	197 N	A
		N	N	703 D	180 D	160 D	200 S	A
E	E	E	E	706 R	183 R	163 R	203 F	A
		F	F	707 Y	184 Y	164 Y	204 Y	A
		F	F	724 Y	201 Y	175 Y	228 L	
			E	736 R	213 M	187 Q	240 D	
			D	739 N	216 N	190 N	243 F	A
			E	740 Q	217 Q	191 Q	244 Q	A
				743 L	220 T	194 I	247 K	A
			E	751 K	228 N	202 H	255 F	
			F	758 Y	235 Y	209 Y	262 L	
			Q	773 E	250 G	224 T	277 N	A
			A	774 S	251 N	225 N	278 N	A
			A	777 S	254 R	228 K	281 D	A
			M	781 E	258 K	232 K	285 T	A
			M	784 R	261 K	235 E	288 F	
			E	785 K	262 M	236 S	289 P	

Figure S2: Comparative sequence analysis of EST1 at positions mutated within this study.
Sequence alignment adapted from [46]. Numbers indicate amino acid positions. Boxes delineate multi-point mutants. Conserved/semi-conserved residues are shaded. [a, GenBank:NP_001164428.1a; b, GenBank:AAO17582b; c, GenBank:NP_963862]. None of the single or multiple point mutations in hEST1A generated in this study, at left, affected the interaction with hTERT a.a. 1-350 when co-expressed in rabbit reticulocyte lysates [51] (data not shown).

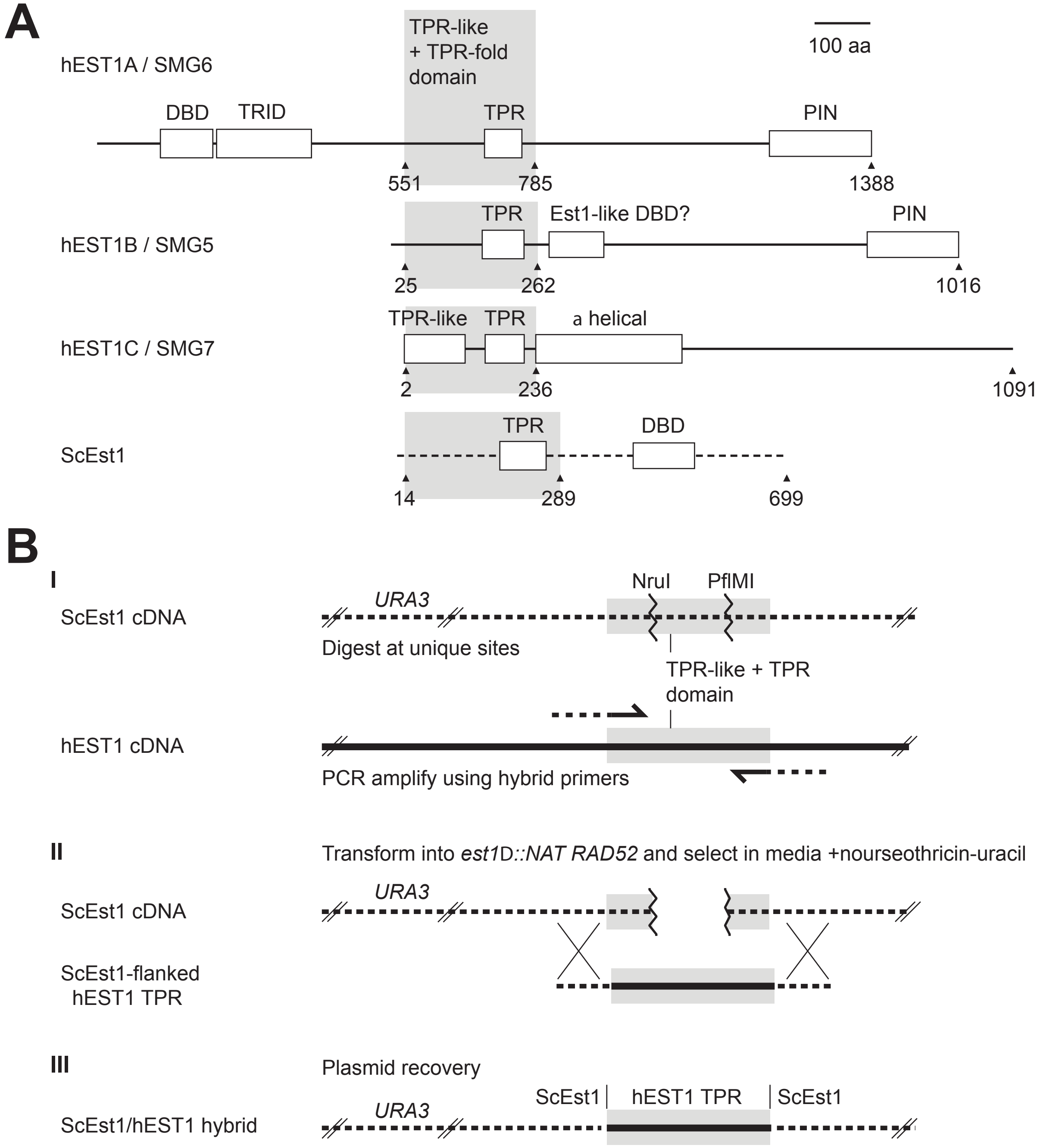

Figure S3: Schematic representation of yeast/human Est1 hybrid proteins.

(A) Shaded areas and amino acid numbers indicate the TPR-containing regions utilized in the construction of yeast/human Est1 hybrid proteins. Boundaries were set according to the structure-based alignment of EST1 sequences [32, 33, 46]. (B) Schematic representation of the *in vivo* gap-repair cloning method used to construct yeast/human Est1 hybrids. Shaded areas represent the TPR domains of ScEst1 and the homologous regions of hEST1A, hEST1B and hEST1C. (I) ScEst1 cDNA (dashed line) was digested in the TPR sequence at two sites that were unique in the plasmid, and treated with phosphatase. The TPR sequence of hEST1 (solid line) was amplified using primers bearing 30-mer hEST1 sequences flanked by 45-mer ScEst1 sequences (refer to Methods). (II) DNA was gel-purified and transformed into *est1Δ::NAT RAD52 S. cerevisiae* (to prevent recombination into the endogenous EST1 locus). Cells were grown in media containing nourseothricin and lacking uracil to select for the *est1Δ* genotype and for regeneration of the URA3 plasmid by homologous recombination. (III) The plasmid, in which the TPR domain sequence of ScEst1 was replaced with that of hEST1, was recovered.

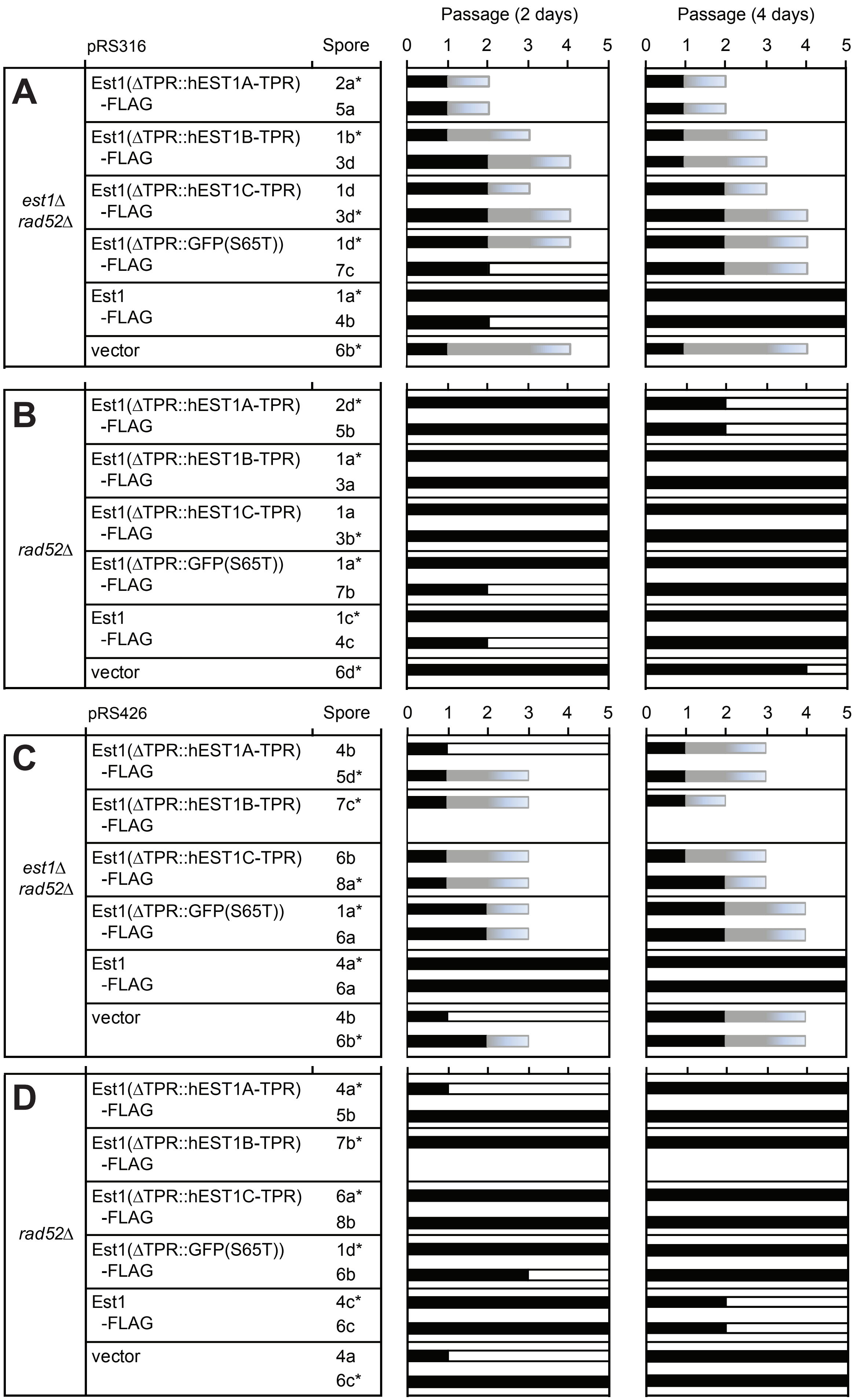

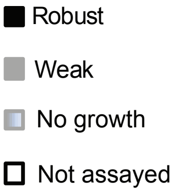

Heterozygous diploid *est1*Δ *rad52*Δ strains were transformed with pRS316(*URA3*) or pRS426(*URA3*) plasmids expressing human/yeast EST1 hybrids in which the TPR domain of ScEst1 was replaced with the TPR domain of human EST1 or GFP(S65T). Haploid spores were isolated and passaged every two or four days on plates containing synthetic dropout media lacking uracil. Cell growth was scored according to the legend. Asterisks (*) indicate spores that were assayed in Figures 2 and 3.

Figure S4: Schematic representation of colony growth in Figure 2. Est1 hybrids do not rescue *est1*Δ *rad52*Δ strains or interfere with growth of *rad52*Δ strains.

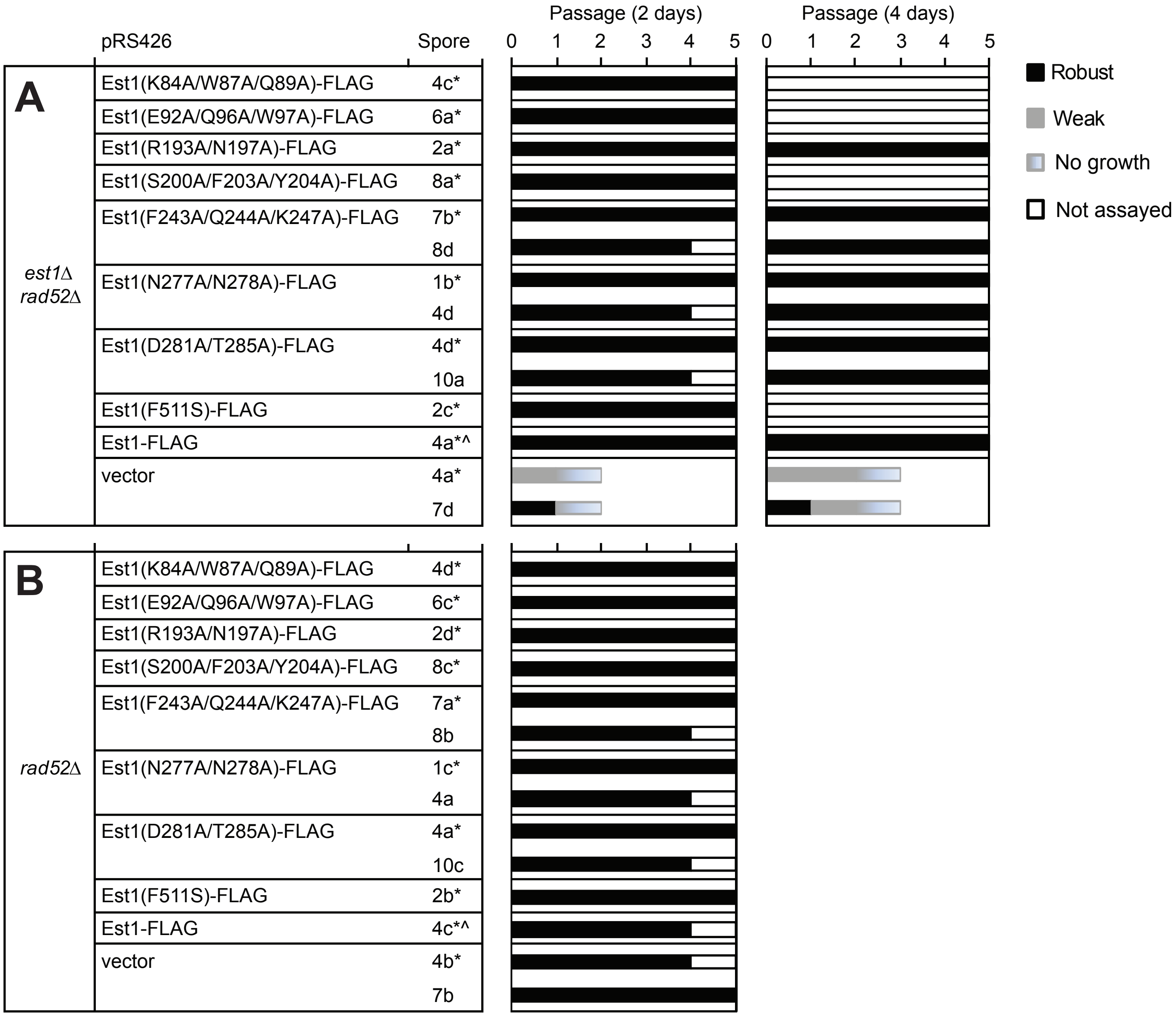

Figure S5: Schematic representation of colony growth in Figure 4. Mutation of the Est1 TPR domain does not compromise viability in *S. cerevisiae*. Heterozygous diploid *est1*Δ *rad52*Δ yeast were transformed with pRS426(*URA3*) plasmids expressing ScEst1 TPR domain mutants. Haploid spores of the indicated genotype were isolated and passaged every two or four days on plates containing synthetic dropout media lacking uracil. Cell growth was scored according to the legend. Asterisks (*) indicate spores that were assayed in Figure 4. ^ indicates the same spores described in Figure 2.

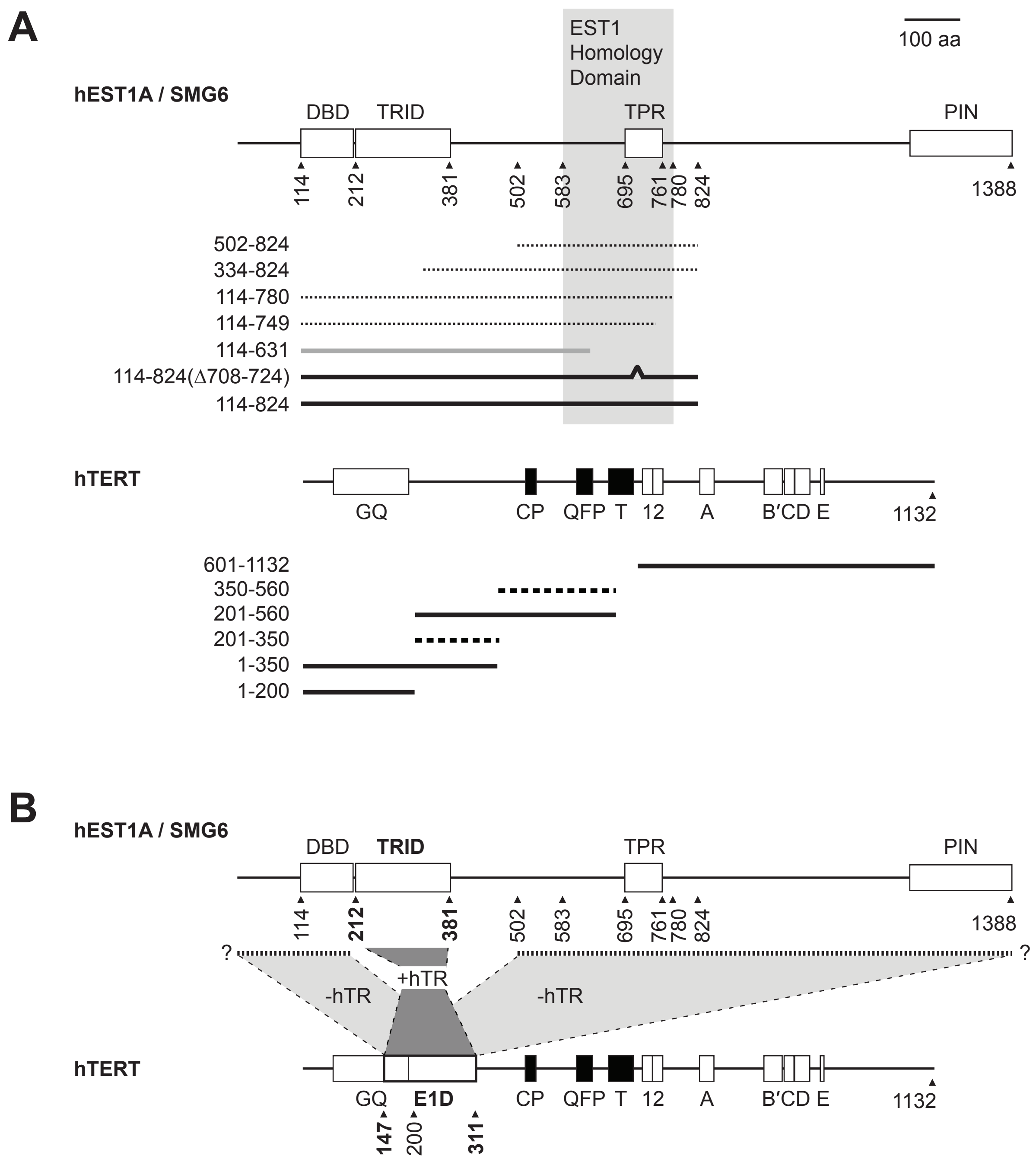

Figure S6: Summary of hTERT/hEST1A co-IP experiments.
(A) Summary of hEST1A TPR domain interactions with the hTERT N- and C-termini (this study). Line representations of hEST1A indicate fragments that precipitated non-specifically *in vitro* (dotted lines). hEST1A(114-631) (grey line) exhibited a weak and non-statistically significant interaction with hTERT (data not shown) [51]. hEST1A(114-824) and hEST1A(114-824,Δ708-724) exhibited interactions with hTERT (dark lines). Line representations of hTERT represent fragments that supported an interaction with hEST1A(114-824) or hEST1A(114-824,Δ708-724) (solid lines), or a non-detectable interaction (dashed lines). (B) Summary of interactions between hEST1A and hTERT described by Redon *et al*. [47]. Redon *et al*. reported an RNA-dependent interaction between an hTR-interaction domain (TRID) of hEST1A and an EST1-interaction domain (E1D) of hTERT. These regions also bind hTR. The authors provided evidence of an RNA-independent interaction of E1D with hEST1A downstream of amino acid 502, although the boundaries of the region were not identified (‘?’). (A, B) Numbers indicate amino acids. hEST1A amino acid numbers correspond to [GenBank:NP_001164428.1]. White/black boxes indicate featured domains.
